# A Tribute to a *Bacillus thuringiensis* Master: Professor David J. Ellar

**DOI:** 10.3390/toxins12120764

**Published:** 2020-12-03

**Authors:** Susana Vílchez

**Affiliations:** Institute of Biotechnology, Department of Biochemistry and Molecular Biology I, Faculty of Science, University of Granada, 18071 Granada, Spain; svt@ugr.es

This Special Issue, on *Bacillus thuringiensis* and its toxins, seems to be the right place to pay tribute to one of the most influential scientists in the field of research into this peculiar bacterium. Professor David J. Ellar passed away at his home in Cambridge, UK, on 21 May 2020, aged 80, as a result of pneumonia, after a long and debilitating illness. Everyone who knew him and had the opportunity to work hand in hand with him remain devastated by his loss.

Professor Ellar developed a very relevant scientific career in the Department of Biochemistry at the University of Cambridge. Through his laboratory, commonly known as Skylab, passed literally hundreds of postdoctoral researchers, visiting professors, PhD students, master’s students, and undergraduate students. We all received the best training possible on *B. thuringiensis*, Cry toxins, their receptors, and above all on how cutting-edge and quality science is conducted. Professor Ellar has been a referent in the field of *B. thuringiensis* for all researchers working with this special bacterium and its entomopathogenic toxins. Thanks to his more than 160 research papers, published in the best scientific journals, we all know today a little bit more about this microorganism, which has proved to be extremely useful in the area of biotechnology. 

Professor Ellar was a pioneer in *B. thuringiensis* research. Thanks to him, we were able to “see” for the first time the three-dimensional structure of a Cry toxin [1]. He was also the first to show us what a Cyt toxin, another entomotoxin produced by *B. thuringiensis*, looked like [2]. Thanks to his research we learned about the existence of the first Cry toxin receptor described [3], present on the enterocyte membrane of an insect, further identified as the well-known APN receptor [4]. This created an opportunity to begin understanding the mechanism of action of Cry toxins. In addition, he was the first to relate the Domain II Loops of a Cry toxin to specificity [5], explaining why some Cry toxins are active against some insects and not against others. He was the first person who managed to successfully display a functional Cry toxin on the surface of a phage [6], opening the possibility of using phage display technology for the in vitro evolution of Cry toxins. 

However, David not only stood out for being a brilliant professional. On a personal level, he was simply a great person. He was always willing to help to anyone in need. He was extremely generous, and he had an outstanding sense of humour. He also had the remarkable ability to keep a huge research group motivated, in which each of its members worked with the precision of a Swiss watch.

I have the pleasure to say that David was a real master for me: the person that I have as a model in life and the person I want to become when I grow older. With this humble letter, and on behalf of the *B. thuringiensis* research community, I would like to give you the most sincere thanks for all your work and knowledge, and for your human greatness. Rest in peace.
